# Compound Liquorice Powder in Diabetes

**Published:** 1909-08-21

**Authors:** 


### COMPOUND LIQUORICE POWDER IN DIABETES.

There is little doubt that the Pulvis Glycyrrhizæ Compositus of the British Pharmacopœia is a very valuable laxative, easy of use, effective, and free from danger. Nevertheless it consists very largely of sugar, so that its use may be contra-indicated, as Dr. Balmanno Squire has pointed out, in a great many cases, notably in diabetes mellitus, gout, and obesity, as the following formulary shows:—

| | | |
|---|---|---|
| Senna ... ... ... | 2 parts | 4 parts. |
| Fennel ... ... ... | 1 part | |
| Sulphur ... ... ... | 1 part | |
| Liquorice ... ... ... | 2 parts | 8 parts. |
| Sugar ... ... ... | 6 parts | |

Dr. Squire has pointed out that those who have attempted to remedy the difficulty have restricted themselves to the simple process of leaving out the sugar and retaining the liquorice, senna, fennel, and sulphur, thereby reducing the dose to half that which is official. The difficulties of this plan, however, are threefold. First, it gives rise to two preparations of the same name differing widely as to dose. Secondly, the amended preparation still contains sweet-stuff—liquorice—to the extent of one-third of its weight. Thirdly, the amended preparation is not at all readily miscible with water, owing to the absence of the sugar. Called after the Christian name of its inventor, Balmanno's powder obviates these difficulties by retaining the two parts of senna and the one part each of fennel and of sulphur, and by replacing the two parts of liquorice and six parts of sugar by eight parts of a powder composed of sweet almonds and gum acacia in the proportion of eight parts of almonds to one of gum. The omission of the liquorice hardly interferes with the laxative effect.

The almonds, having first been blanched and dried, are triturated in a mortar to a fine powder. The gum, likewise powdered, and the senna, fennel, and sulphur are then added, and the product is a very presentable preparation that is far less mawkish to the taste than is pulv. glycyrrhizæ co., is more miscible with water, has the same dosage, and yet contains no sugar that might be deleterious in a diabetic case.

